# Platycodin D from *Platycodonis Radix* enhances the anti-proliferative effects of doxorubicin on breast cancer MCF-7 and MDA-MB-231 cells

**DOI:** 10.1186/1749-8546-9-16

**Published:** 2014-06-09

**Authors:** Zheng-Hai Tang, Ting Li, Hong-Wei Gao, Wen Sun, Xiu-Ping Chen, Yi-Tao Wang, Jin-Jian Lu

**Affiliations:** 1 State Key Laboratory of Quality Research in Chinese Medicine, Institute of Chinese Medical Sciences, University of Macau, Macao, China

## Abstract

**Background:**

It has been demonstrated that platycodin D (PD) exhibits anti-cancer activities. This study aims to investigate the anti-proliferative effects of the combination of PD and doxorubicin (DOX) on human breast cancer cells (MCF-7 and MDA-MB-231 cells).

**Methods:**

The anti-proliferative effects of different dosages of PD, DOX, and PD + DOX on MCF-7 and MDA-MB-231 cells were determined by the MTT assay. The 10 μM PD, 5 μM DOX, and 10 μM PD + 5 μM DOX induced-protein expression of apoptosis-related molecules on MCF-7 and MDA-MB-231 cells were detected by western blot. The 10 μM PD, 5 μM DOX and 10 μM PD + 5 μM DOX-induced mitochondrial membrane potential changes on MCF-7 and MDA-MB-231 cells were stained with JC-1 before visual determination. The intracellular accumulations of DOX, induced by 10 μM PD, 5 μM DOX and 10 μM PD + 5 μM DOX, were detected by flow cytometry.

**Results:**

PD enhanced anti-cancer activities of DOX were observed in both MCF-7 and MDA-MB-231 cell lines. Compared with mono treatment, the combined treatment increased the protein expression of cleaved poly (ADP-ribose) polymerase and decreased the mitochondrial membrane potential. The combined treatment with PD did not obviously increase the accumulation of DOX in MCF-7 cells (1.66 ± 0.13 in DOX-treated group, and 1.69 ± 0.06 in PD + DOX-treated group, *P* = 0.76), but it significantly increased the accumulation of DOX in MDA-MB-231 cells (1.76 ± 0.17 in DOX-treated group, 2.09 ± 0.02 in PD + DOX-treated group, *P* = 0.027).

**Conclusion:**

The combined treatment of DOX and PD exhibited stronger anti-proliferative effects on MCF-7 and MDA-MB-231 cells than DOX and PD treatment did.

## Introduction

Breast cancer is one of the major lethal cancers in women worldwide; more than 200,000 individuals have been diagnosed with breast cancer in the United States in 2012 [1]. The clinical therapeutic regimens for breast cancer include resection, chemotherapy, radiotherapy and biological therapy [2]. However, the treatments of breast cancer are commonly unsuccessful, because of its secondary recurrence, metastasis and drug resistance [3,4]. The chemotherapy drug doxorubicin (DOX), which is an anthracycline anti-cancer drug, has been widely used to treat breast cancer, leukemia and lung cancer by inhibiting DNA topoisomerase II and intercalating double strand DNA [5,6]. In breast cancer therapy, DOX has been the preferred drug for more than four decades after its discovery [7]. However, DOX has cardiotoxicity [8]. Therefore, combined use with other compounds to reduce the dosage and cardiotoxicity of DOX without affecting its anti-cancer effect would be desirable.

The herb *Platycodonis Radix*, which has been used for chronic inflammatory diseases for centuries in China, contains platycodin D (PD) as one of its main active constituents [9]. PD exhibited anti-inflammatory, anti-allergic, cholesterol-lowering and neuroprotective properties [10,11], and exerted remarkable anti-cancer effects on different kinds of cancer cell lines, such as HepG2, MDA-MB-231, U937, K562, and THP-1 *etc.*, by promoting apoptosis, inducing cell cycle arrest, and inhibiting the migration and invasion of cancerous cells [12-15]. Reducing the telomerase activity [13], increasing reactive oxygen species [16], suppressing the epidermal growth factor receptor-mediated Akt and mitogen-activated protein kinase activation [14], and activating the caspase pathway [17] were suggested involved in the anti-cancer mechanism of PD.

This study aims to investigate the anti-proliferative effects of the combination of PD and DOX on MCF-7 and MDA-MB-231 cells.

## Methods

### Reagents

DOX and dimethyl sulfoxide (DMSO) were obtained from Sigma (St. Louis, MO, USA). DOX was dissolved in DMSO at a concentration of 40 mM, and stored at −20°C. PD was purchased from Chengdu Best Reagent Co., LTD (Chengdu, Sichuan, China), and dissolved in DMSO at a concentration of 40 mM and stored at −20°C. 3-[4,5-Dimethyl-2-thiazolyl]-2,5-diphenyltetrazolium bromide (MTT) and 5,5′,6,6′-tetrachloro-1,1′,3,3′-tetraethylbenzimi-dazolylcarbocyanine iodide (JC-1) were purchased from Molecular Probes (Eugene, OR, USA). RPMI 1640 medium, fetal bovine serum (FBS) and antibiotics were purchased from Gibco (Carlsbad, CA, USA). Primary antibodies [*i.e.*, poly (ADP-ribose) polymerase (PARP) and β-actin], and secondary antibodies were obtained from Cell Signaling Technology (Beverly, MA, USA).

### Cell line and culture

MCF-7 (ATCC number: HTB-22) and MDA-MB-231 (ATCC number: HTB-26) human breast cancer cell lines were obtained from the American Type Culture Collection (ATCC, Rockville, MD, USA). The cells were cultured in a RPMI 1640 medium supplemented with 10% (v/v) FBS and antibiotics (100 U/mL penicillin, 100 μg/mL streptomycin). The cells were grown in a 5% CO_2_ incubator at 37°C.

### MTT assay

The effects of DOX (0.3125 μM, 0.625 μM, 1.25 μM, 2.5 μM, and 5 μM), PD (1.25 μM, 2.5 μM, 5 μM, 10 μM, and 20 μM), and DOX + PD (0.3125 μM DOX + 1.25 μM PD, 0.625 μM DOX + 2.5 μM PD, 1.25 μM DOX + 5 μM PD, 2.5 μM DOX + 10 μM PD, and 5 μM DOX + 20 μM PD) on MCF-7 and MDA-MB-231 cell proliferation were examined by the MTT assay [18]. Exponentially growing MCF-7 and MDA-MB-231 cells were seeded onto 96-well plates. Upon reaching approximately 70% to 80% confluence, the cells were incubated with the indicated compounds for 48 h. Cell viability was determined by incubating the cells in a medium containing 1 mg/mL MTT for 4 h. Then, 100 μL of DMSO was added to solubilize the formazan by shaking for 10 min in the dark. The absorbance at 570 nm was recorded with a microplate reader (Perkin Elmer, 1420 Multilabel Counter Victor3, Wellesley, MA, USA).

### Observation of morphological changes

Exponentially growing MCF-7 and MDA-MB-231 cells were seeded onto 6-well plates. After adhesion, cells were treated with 2.5 μM DOX, 10 μM PD or 2.5 μM DOX + 10 μM PD for 24 h. Then, the cellular morphology was observed with an AxioCam HRC CCD camera (Carl Zeiss, Germany).

### Western blot analysis

MCF-7 and MDA-MB-231 cells were treated with and without 2.5 μM DOX, 10 μM PD, and 2.5 μM DOX + 10 μM PD for 24 h. Total protein was extracted with a radioimmunoprecipitation lysis buffer containing 1% phenylmethanesulfonyl fluoride and 1% protease inhibitor cocktail for 25 min. The protein concentrations were determined with the BCA™ Protein Assay Kit (Pierce, Rockford, IL, USA). Equal amounts of proteins were separated by sodium dodecyl sulfate-polyacrylamide gel electrophoresis and transferred to a PVDF membrane followed by blocking in 5% non-fat dried milk for 1 h. The membrane was incubated with specific primary antibodies against PARP (1:1000) and β-actin (1:2000) followed by incubation with the corresponding secondary antibodies. The specific protein bands were visualized with an ECL advanced western blot analysis detection kit (BD Biosciences, Bedford, MA, USA).

### Mitochondrial membrane potential assay by JC-1 staining

The mitochondrial membrane potential (MMP) assay of intact cells was stained with JC-1 before visual determination [19]. JC-1 probe is a dual-emission fluorescent dye that can reflect the changes in MMP. It forms aggregates that result in a red emission in normal polarized mitochondria, while it forms monomers that emit green fluorescence on the depolarized mitochondrial membrane, the MMP depolarized is an early phenomenon of apoptosis [20]. MCF-7 and MDA-MB-231 cells were seeded onto 96-well plates. After a 24 h incubation for adhesion, the cells were treated with the indicated compounds for 4 h. The medium was removed and the cells were incubated with JC-1 probe for 30 min. Then, the medium with the probe was removed and the cells were rinsed with phosphate-buffered saline (PBS). The cells were observed with a fluorescent microscope, and images were obtained with an Axiovert 200 fluorescent inverted microscope (Carl Zeiss) and an AxioCam HRc CCD camera (Carl Zeiss).

### DOX uptake detection

The intracellular uptake of DOX was examined as previously described [21]. Briefly, MCF-7 and MDA-MB-231 cells were seeded onto 12-well plates. After adhesion, the cells were treated with and without the indicated compounds for 1 h. The cells were then washed with PBS three times and re-suspended in PBS. The mean fluorescence intensities of the cells were determined with a fluorescence-activated cell sorting (FACS) cytometer (FACSCalibur, Becton Dickinson, San Jose, CA, USA).

### Statistical analysis

All experiments were repeated at least three times. The mean ± standard deviation (SD) was determined for each group. Statistical analysis was performed with one-way analysis of variance (one-way ANOVA) and Tukey’s test. Differences were considered statistically significant when *P* < 0.05, and the exact *P* values were shown unless *P* < 0.001. The concentration dependence was visually determined from the graphs.

## Results

### PD enhanced the inhibition of cell proliferation by DOX

**Figure 1:**
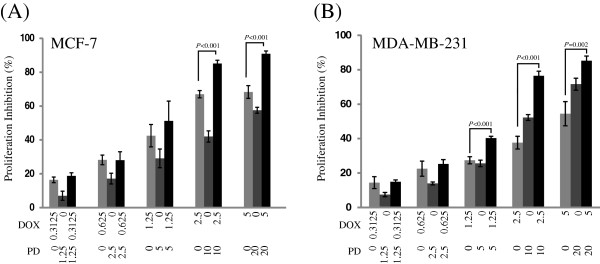
**Effects of DOX, PD and DOX + PD on the viability of breast cancer cells.** MCF-7 **(A)** and MDA-MB-231 **(B)** breast cancer cells were treated with various concentrations of DOX, PD or DOX + PD for 48 h. Then, the cell viability was determined by the MTT assay. Values were expressed as mean ± SD of three independent assays. Statistical analysis was performed with one-way ANOVA and Tukey’s test.

The anti-proliferative effect of DOX, PD and DOX + PD on the human breast cancer cell lines, MCF-7 and MDA-MB-231, was shown in Figure 1. The DOX, PD, and DOX + PD displayed cytotoxicity in a concentration-dependent manner in both cell lines. At low concentrations of DOX + PD groups (0.3125 μM DOX + 1.25 μM PD, and 0.625 μM DOX + 2.5 μM PD), PD did not significantly enhance the cytotoxic characteristic of DOX in MCF-7 (*P* = 0.06 and 0.96, respectively) and MDA-MB-231 (*P* = 0.82 and 0.40, respectively) cells. In the 1.25 μM DOX + 5 μM PD group, the treatment of DOX + PD also did not significantly increase the cytotoxicity of DOX in MCF-7 cells (*P* = 0.18), whereas the anti-proliferative effect of DOX in MDA-MB-231 cells was significantly enhanced (*P* < 0.001). In addition, a significantly enhanced effect was observed in the high concentration of DOX + PD treatment groups (2.5 μM DOX + 10 μM PD and 5 μM DOX + 20 μM PD) in MCF-7 (*P* < 0.001 and *P* < 0.001, respectively) and MDA-MB-231 cells (*P* < 0.001 and *P* = 0.002, respectively).

### PD facilitated DOX-induced morphologic changes

**Figure 2:**
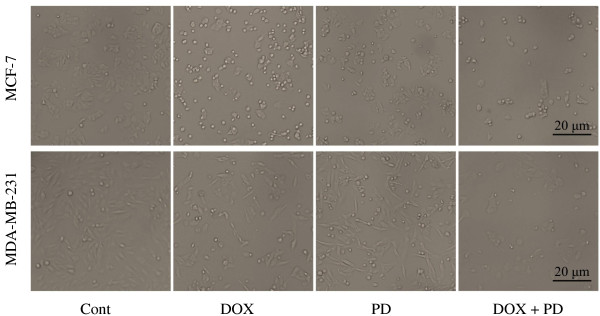
**Morphological changes in breast cancer cells after DOX, PD, and DOX + PD treatments.** MCF-7 and MDA-MB-231 cells were treated with 5 μM DOX, 10 μM PD, or 5 μM DOX + 10 μM PD. After 24 h of treatment, the cell morphological changes were observed with an inverted-phase contrast microscope.

Considering the combined treatment results, we used 2.5 μM DOX + 10 μM PD for further studies. In MCF-7 cells, rounder cells and lower cell numbers were observed in the 2.5 μM DOX +10 μM PD treatment groups compared with that of the control group, and this phenomenon was more obvious in the combined treatment group. Similar to the results of MCF-7 cells, significantly lower cell numbers and rounder cells were observed in the combined treatment group than in the mono treatment groups in MDA-MB-231 cells (Figure 2).

### PD sensitized DOX-triggered apoptosis

**Figure 3:**
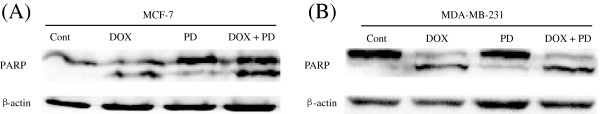
**Effects of DOX, PD, and DOX + PD on PARP protein expression in breast cancer cells.** MCF-7 **(A)** and MDA-MB-231 **(B)** cells were incubated with 5 μM DOX, 10 μM PD, or 5 μM DOX + 10 μM PD for 24 h. Then, the cells were collected and their PARP protein expression was measured by western blot analysis.

Apoptosis is a major cause of cancer cell growth inhibition and is characterized by the up-regulation of the cleaved-PARP protein expression [22]. Herein, we determined the effects of 2.5 μM DOX, 10 μM PD and the combined treatment on the protein expression of PARP through western blot analysis (Figure 3). In MCF-7 cells, the 2.5 μM DOX, 10 μM PD and the combined treatments increased the protein expression of cleaved-PARP. This increased effect was the strongest in the combined treatment group. In MDA-MB-231 cells, the combined treatment of 2.5 μM DOX + 10 μM PD for 24 h also resulted in a higher protein expression of cleaved-PARP than that in the mono treated cells.

### PD increased DOX-induced MMP changes

**Figure 4:**
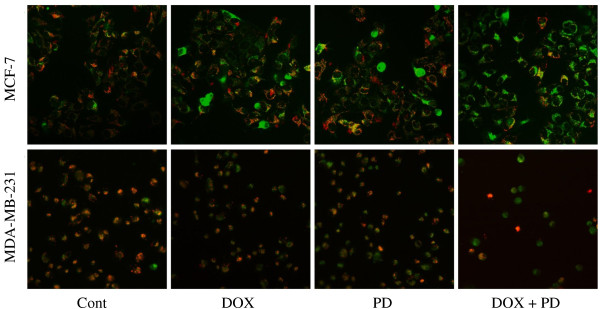
**JC-1 staining of breast cancer cells after DOX, PD, and DOX + PD treatments.** MCF-7 and MDA-MB-231 cells were treated with 5 μM DOX, 10 μM PD, or 5 μM DOX + 10 μM PD. After 4 h of treatment, JC-1 fluorescence was utilized to study the mitochondrial membrane potential change. The cells were observed with a fluorescent microscope, and images were obtained with an Axiovert 200 fluorescent inverted microscope and an AxioCam HRc CCD camera.

Given that MMP has a critical effect on cell apoptosis [23], we determined the MMP changes after DOX, PD, or DOX + PD treatments for 4 h in both MCF-7 and MDA-MB-231 cell lines. As shown in Figure 4, the 2.5 μM DOX and 10 μM PD treatments slightly enhanced the green fluorescence in MCF-7 cells, compared with that in the control group. However, the green fluorescence was remarkably enhanced in the DOX + PD treatment group. The MMP changes in MDA-MB-231 cells were similar to those in MCF-7 cells.

### Intracellular DOX uptake

**Figure 5:**
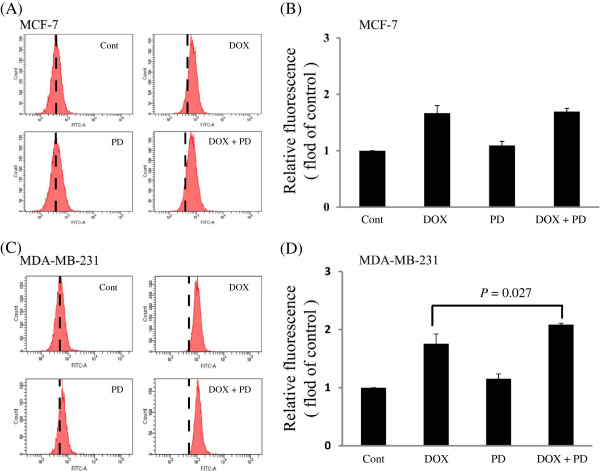
**Intracellular DOX accumulation in breast cancer cells.** Cells were pretreated with 5 μM DOX, 10 μM PD, or 5 μM DOX + 10 μM PD for 1 h. Then, the cells were washed, harvested, and re-suspended in PBS. Intracellular fluorescence was measured by flow cytometry analysis. The fluorescence intensity indicates the intracellular DOX concentration. **(A)** Flow cytometry assay images of MCF-7 cells; **(B)** statistical results of three independent tests of A; **(C)** flow cytometry assay images of MDA-MB-231 cells; and **(D)** statistical results of three independent tests of C. Values were expressed as mean ± SD of three independent assays. Statistical analysis was performed with one-way ANOVA and Tukey’s test.

The accumulation of DOX in MCF-7 and MDA-MB-231 cells was examined by the FACS assay, to determine if PD enhances the cytotoxicity effect of DOX by increasing the intracellular concentration of DOX. Given that DOX exhibits self-fluorescence, the mean intensity of the intracellular fluorescence was used to reflect the intracellular concentration. In MCF-7 cells, the combined treatment of PD + DOX did not obviously enhance the intracellular concentration of DOX, while in MDA-MB-231 cells, the intracellular DOX concentration in the combined treatment group was obviously higher compared with that in the DOX alone treatment group (Figure 5).

## Discussion

Numerous studies have demonstrated that combined treatment with Chinese herbs to enhance the anti-cancer properties and reduce the side effects of chemotherapy drugs is a new strategy for cancer therapy [24,25]. In this study, the combined treatment of DOX + PD exerted a stronger inhibitory effect on the growth of MCF-7 and MDA-MB-231 cells than the individual treatments did. The relatively low DOX and PD concentration groups exhibited a slightly increased anti-proliferative effect, while the high concentration groups presented an obvious elevated anti-proliferative effect. In addition to the present concentration ratio of DOX and PD (DOX:PD = 1:4), we tested another concentration ratio of DOX and PD (DOX:PD = 1:16) in MCF-7 cells and found that the combined treatment also exhibited a higher anti-proliferative effect than the mono treatment did (Additional file 1). Collectively, PD could enhance the anti-proliferative effect of DOX and might decrease the cardiotoxicity and clinical dosage of DOX without affecting its anti-cancer effect.

Breast cancer can be divided into two types, estrogen receptor (ER) negative and ER positive; the MCF-7 cell line is ER positive, whereas the MDA-MB-231 cell line is ER negative [26]. At relatively high concentrations of the combined treatment, the PD-enhanced DOX-induced anti-proliferative effect was more remarkable in the MDA-MB-231 cell line than in the MCF-7 cell line. The intracellular DOX uptake in the combined treatment group was obviously higher than that in the DOX group in MDA-MB-231 cells. This result indicates that PD partially enhanced the anti-proliferative effect of DOX by increasing the intracellular concentration of DOX. However, the intracellular DOX uptake in the combined treatment and DOX groups showed no remarkable difference in MCF-7 cells, indicating that the combined anti-proliferative effect and intracellular DOX accumulation were different between two cancer cell lines and cancer types.

Apoptosis has a critical effect on cancer treatment, and up-regulation of apoptosis is one of the anti-growth methods in cancer therapy [27]. The combined therapy of DOX + PD had a higher apoptotic effect than the individual treatments in both the MCF-7 and MDA-MB-231 cell lines. This enhancement might be one of the reasons for the enhanced anti-proliferative effect of the combined treatment. Besides, the combined treatment group exhibited a higher depolarized MMP than the mono treatment did in both the MCF-7 and MDA-MB-231 cell lines, indicating that the enhanced apoptotic effect in the combined group might be caused by increased depolarization of MMP.

The combined treatment of PD + DOX had a stronger anti-growth effect than single-agent therapy does in MCF-7 and MDA-MB-231 cells. The enhanced effect might partially be caused by sensitized apoptosis. PD may be employed as an adjuvant therapy for breast cancer cells when DOX is used.

## Conclusions

The combinative treatment of DOX and PD exhibited stronger anti-cancer effectiveness than DOX and PD treatment alone in MCF-7 and MDA-MB-231 cells.

## Abbreviations

ATCC: American type culture collection; DMSO: Dimethyl sulfoxide; DOX: Doxorubicin; ER: Estrogen receptor; FACS: Fluorescence-activated cell sorting; FBS: Fetal bovine serum; JC-1: 5,5′,6,6′-tetrachloro-1,1′,3,3′-tetraethyl-benzimidazolylcarbocyanine iodide; MTT: 3-[4,5-dimethyl-2-thiazolyl]-2,5-diphenyl tetrazolium bromide; MMP: Mitochondrial membrane potential; one-way ANOVA: One-way analysis of variance; PARP: Poly (ADP-ribose) polymerase; PBS: Phosphate-buffered saline; PD: Platycodin D; SD: Standard deviation.

## Competing interests

The authors declare that they have no competing interests.

## Authors’ contributions

ZHT, TL, YTW and JJL conceived and designed the experiments. ZHT, TL, HWG and WS performed the experiments. ZHT, TL, XPC and JJL analyzed the data. ZHT and JJL wrote the manuscript. All authors read and approved the final version of the manuscript.

## Supplementary Material

Additional file 1 **Combined treatment (DOX: PD = 1: 16) exhibited a higher anti-proliferative effect than the mono treatment did.** Effects of DOX, PD and DOX+PD on the viability of MCF-7 cells. MCF-7 breast cancer cells were treated with various concentrations of DOX, PD or DOX+PD for 48 h. Then, the cell viability was determined by the MTT assay. Values were expressed as mean ± SD of three independent assays. Statistical analysis was performed with one-way ANOVA and Tukey’s test. Click here for file
