# Supplementary material for: Platycodin D from Platycodonis Radix enhances the anti-proliferative effects of doxorubicin on breast cancer MCF-7 and MDA-MB-231 cells
Source: Chin Med. 2014 Jun 9;9:16. doi: 10.1186/1749-8546-9-16 (PMC4075934; doi:10.1186/1749-8546-9-16)
Supplement: Additional file 1 — Combined treatment (DOX: PD = 1: 16) exhibited a higher anti-proliferative effect than the mono treatment did. Effects of DOX, PD and DOX+PD on the viability of MCF-7 cells. MCF-7 breast cancer cells were treated with various concentrations of DOX, PD or DOX+PD for 48 h. Then, the cell viability was determined by the MTT assay. Values were expressed as mean ± SD of three independent assays. Statistical analysis was performed with one-way ANOVA and Tukey’s test. [file 1749-8546-9-16-S1.pdf]

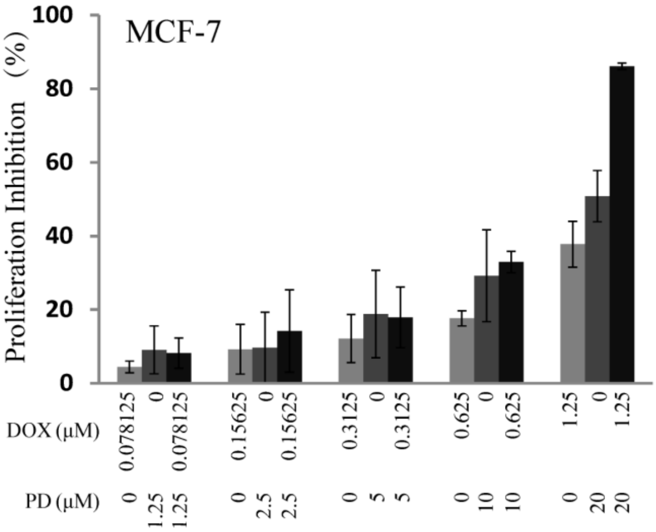

Supplementary file 1. Effects of DOX, PD and DOX + PD on the viability of MCF-7 cells. MCF-7 breast cancer cells were treated with various concentrations of DOX, PD or DOX + PD for 48 h. Then, the cell viability was determined by the MTT assay. Values were expressed as mean ± SD of three independent assays.
